# Burden of medically attended influenza in Norway 2008‐2017

**DOI:** 10.1111/irv.12627

**Published:** 2019-01-13

**Authors:** Siri Helene Hauge, Inger Johanne Bakken, Birgitte F. de Blasio, Siri E. Håberg

**Affiliations:** ^1^ Division of Infection Control and Environmental Health Norwegian Institute of Public Health Oslo Norway; ^2^ Centre for Fertility and Health Norwegian Institute of Public Health Oslo Norway; ^3^ Department of Biostatistics Oslo Centre for Biostatistics and Epidemiology Institute of Basic Medical Sciences University of Oslo Oslo Norway

**Keywords:** disease outbreak, hospitalization, influenza, human, Norway, pandemics

## Abstract

**Background:**

The burden of influenza in Norway remains uncertain, and data on seasonal variations and differences by age groups are needed.

**Objective:**

To describe number of patients diagnosed with influenza in Norway each season and the number treated in primary or specialist health care by age. Further, to compare the burden of seasonal influenza with the 2009‐2010 pandemic outbreak.

**Methods:**

We used Norwegian national health registries and identified all patients diagnosed with influenza from 2008 to 2017. We calculated seasonal rates, compared hospitalized patients with patients in primary care and compared seasonal influenza with the 2009‐2010 pandemic outbreak.

**Results:**

Each season, on average 1.7% of the population were diagnosed with influenza in primary care, the average rate of hospitalization was 48 per 100 000 population while the average number of hospitalized patients each season was nearly 2500. The number of hospitalized influenza patients ranged from 579 in 2008‐2009 to 4973 in 2016‐2017. Rates in primary care were highest among young adults while hospitalization rates were highest in patients 80 years and older and in children below 5 years. The majority of in‐hospital deaths were in patients 70 years and older. Fewer patients were hospitalized during the 2009‐2010 pandemic than in seasonal outbreaks, but during the pandemic, more people in the younger age groups were hospitalized and fatal cases were younger.

**Conclusion:**

Influenza causes a substantial burden in primary care and hospitals. In non‐pandemic seasons, people above 80 years have the highest risk of influenza hospitalization and death.

## 1 INTRODUCTION

Even though infectious diseases have become less important as cause of morbidity and mortality in middle‐ and high‐income countries, yearly influenza outbreaks remain a major cause of sick leave, doctor visits, hospitalizations and deaths worldwide.^1^, ^2^, ^3^, ^4^, ^5^, ^6^, ^7^ Influenza outbreaks occur every winter in temperate climate zones. A UK study found that 18% of the unvaccinated population was infected each season.^8^ Studies from several countries support that hospitalizations and complications of influenza are substantial.^9^, ^10^, ^11^, ^12^, ^13^ For children under 5 years of age, it has been estimated that influenza contributes to around 870 000 hospitalizations each year globally.^3^ Among the elderly, hospitalization rates and deaths are higher than in younger age groups.^4^, ^6^ A recent study suggested that seasonal influenza outbreaks cause up to 645 000 deaths each year globally.^6^ The yearly number of fatalities in Norway has been estimated to be around 900.^14^ In pandemic outbreaks, younger age groups are more affected and have higher hospitalization rate than the elderly.^15^

The yearly outbreak of influenza in Norway, with a population size of around 5 million people, costs more than 250 million US $ (2005 US$), including costs for sick leave, medications, doctors’ visits and hospitalizations.^13^ Information on the disease burden of influenza is of value for national authorities when making policies on disease prevention, vaccine recommendations and hospitals in planning and scaling their capacity. Better data on severe influenza will inform physicians on the risk associated with influenza infection, which is useful when advising patients on preventive measures such as vaccination. Vaccination coverage, even in groups recommended seasonal influenza vaccination, is low in Europe, including Norway.^16^, ^17^, ^18^

Many countries still lack good surveillance systems and have little information on the burden of influenza.^19^ In Norway, national health registries provide data from the complete population, which makes it possible to study the burden of influenza on the healthcare system at a national level.

We aimed to describe the burden of medically attended influenza in Norway in the period 2008‐2017. We utilized primary care data, emergency visits data and hospital data to investigate age effects and the overall impact of influenza in primary and hospital care. We also compared the 2009‐10 pandemic outbreak to seasonal influenza outbreaks in terms of the age distribution of patients affected.

## 2 METHODS

### 2.1 Study population and study period

Our study population consisted of the total Norwegian population, and we included all persons registered as residents between 2008 and 2017. We retrieved the total population number per January 1 each year from Statistics Norway. The study period was from January 1, 2008, to December 31, 2017. During this period, the Norwegian population increased from 4 737 171 to 5 258 317.^20^

### 2.2 Data sources

Our study is based on prospectively collected data from primary care and hospitals. Data were linked using unique personal identification numbers ensuring correct linkage at the individual level. Population data were retrieved from Statistics Norway (SSB). The Regional Ethical Committee approved the study.

### 2.3 Primary care data

All inhabitants in Norway are designated to a general practitioner (GP), and 99.3% of the population is enrolled in this public primary care system.^21^ The Directorate of Health's system for control and payment of health reimbursements receives claims from all publicly funded GPs and primary care emergency clinics in Norway. From this database, we collected data on age, sex and date of diagnosis for patients who were diagnosed with influenza using the registry code R80 in the International Classification of Primary Care (ICPC‐2) diagnostic system. This diagnosis is based on clinical criteria and does not require laboratory confirmation.^22^ We included both face‐to‐face consultations and consultations by phone.

### 2.4 Hospital data

The Norwegian Patient Registry (NPR) holds clinical data from all hospitals in Norway.^23^ Reporting to the NPR is mandatory and includes diagnoses according to the International Classification of Diseases (ICD‐10). We retrieved date of hospitalization and vital status at discharge on all patients diagnosed with the ICD‐10 codes J09‐J11 (influenza) in the years 2008‐2017. While most of the J09‐J11 codes require laboratory confirmation, information on laboratory testing is not reported to the NPR.

### 2.5 Influenza seasons

We defined the influenza seasons according to the surveillance period which follow the standard of the European Centre for Disease Control (ECDC).^24^ Thus, an influenza season was defined as the period from late September or beginning of October (calendar week 40) until May the following year (calendar week 20), with an exception of 2009 where the pandemic season included twelve months from May 2009 onwards. Patients diagnosed outside the defined seasons were not included in the analysis unless otherwise specified. For patients diagnosed more than once within a 120‐day interval, we included the first encounter in that season only, except in some analyses where we specify other inclusions. Our study period covers nine consecutive influenza seasons, from the 2008‐2009 season to the 2016‐2017 season also including the 2009‐2010 pandemic outbreak.

### 2.6 Analysis

We list the number and proportions of the population diagnosed with influenza each season in primary care and hospitals. We calculated seasonal rates by dividing number of cases in the defined period by the total population number for the corresponding year. For age‐specific rates, we used number of persons in each age group. We used negative binomial regression analyses to compare risks between age groups for influenza diagnosis in primary care or hospitals, using the age groups 40‐59 years as reference. For statistical analysis, we used Stata (StataCorp. 2017. Stata Statistical Software: Release 15. College Station, TX: StataCorp LLC).

## 3 RESULTS

**Figure 1:**
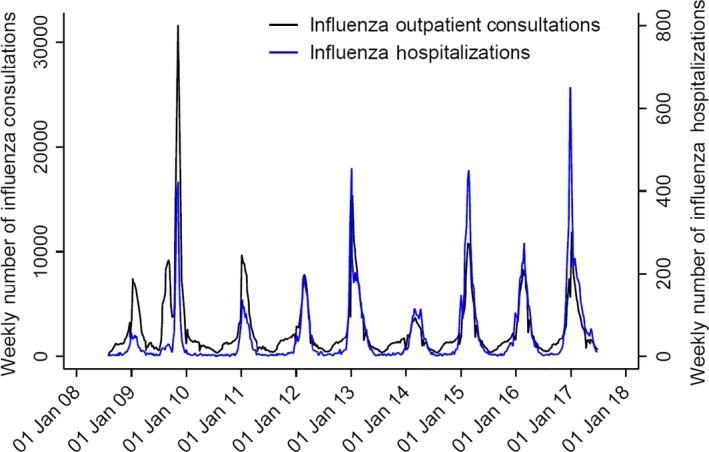
Weekly number of influenza diagnoses in primary care and hospitals in Norway in the period 2008‐2017. Numbers include all consultations, also multiple hospitalizations and influenza diagnoses within one season, and diagnoses made outside defined seasons

Influenza activity showed a clear seasonal pattern, and all seasonal outbreaks peaked in January, February or March. The 2009‐2010 pandemic outbreak was different and peaked in November 2009. The timing of the diagnoses for primary care and hospitals mostly coincided (Figure 1). The number of patients diagnosed varied largely between seasons (Table 1).

**Table 1:** Number of patients^a^ in primary care, hospitalizations and fatal cases with influenza diagnoses in Norway, 2018‐2017

Influenza season	Patients in primary care (% of total population)	Hospitalizations (per 100 000 population)	Number of fatal cases in hospitals (average age)	Proportion of hospitalized patients with fatal outcome	Number of days with hospital care^b^
2008‐2009	68 919 (1.4)	579 (12)	8 (49)	1.4	2572
2009‐2010	189 875 (3.9)	2048 (42)	28 (47)	1.4	9100
2010‐2011	80 072 (1.6)	1157 (24)	17 (62)	1.5	6301
2011‐2012	76 041 (1.5)	1750 (35)	36 (80)	2.1	10 069
2012‐2013	108 197 (2.1)	3314 (66)	85 (76)	2.6	19 267
2013‐2014	49 510 (1.0)	1517 (30)	47 (75)	3.1	9537
2014‐2015	91 377 (1.8)	3915 (76)	124 (82)	3.2	23 412
2015‐2016	80 771 (1.6)	2975 (57)	88 (70)	3.0	17 479
2016‐2017	92 462 (1.8)	4973 (95)	232 (81)	4.7	33 118
Average of all nine seasons	93 025 (1.7)	2470 (48)	74 (76)	3.0	14 943

a Not including patients with multiple registrations in the same influenza season.

b Including patients with multiple hospitalizations in the same influenza season.

### 3.1 Influenza burden in primary care

During the nine influenza seasons, a total of 1 099 723 influenza diagnoses were registered in primary care, giving an average of 122 191 consultations with influenza patients each season. Multiple registrations for the same person within a 120‐day interval in the same season were considered to represent one episode of influenza infection. After removing multiple registrations within 120 days, there were 837 224 patients which we included in further analysis, of whom 55% were female. The average age was 36 years (median also 36). Number of patients in each season ranged from 68 919 in 2008‐2009 to 189 875 in 2009‐2010 (Table 1). On average, 1.7% of the population were diagnosed with influenza during an outbreak; however, it varied from 1.0% during the 2013‐2014 season to 3.9% during the 2009‐2010 pandemic (Table 1).

**Figure 2:**
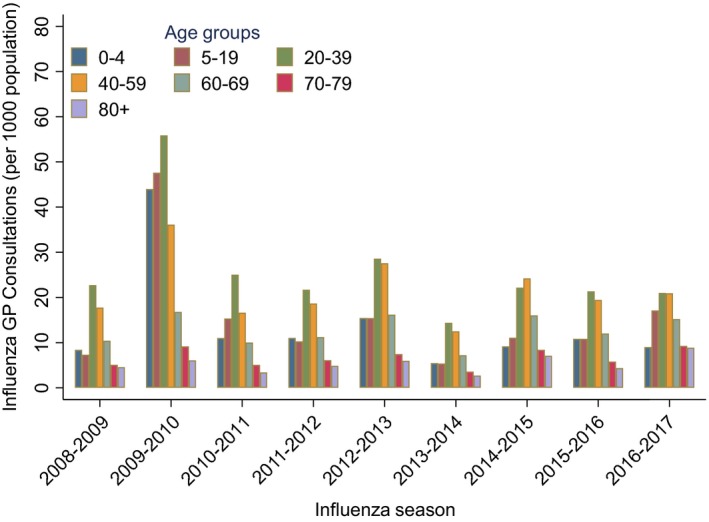
Seasonal age‐specific rates of primary care patients diagnosed with influenza (per 1000 population), in Norway during 2008‐2017

**Figure 3:**
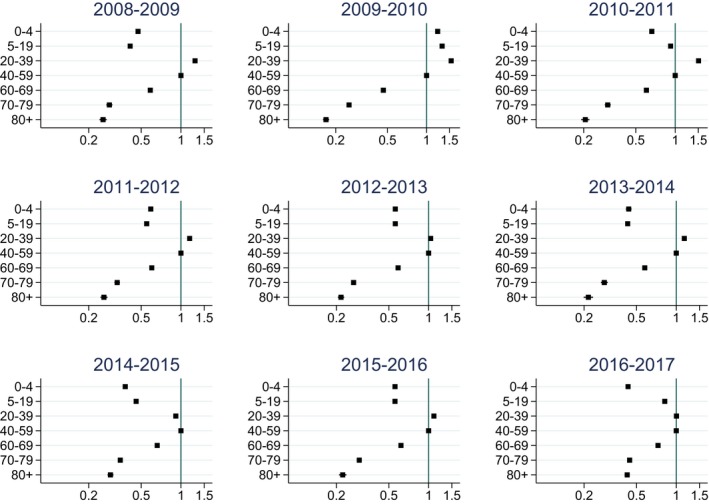
Risk ratios (with 95% confidence intervals) for being diagnosed with influenza in primary care by age group in each influenza season. Logarithmic scale on *x*‐axis. Reference age group: 40‐59 years

Primary care rates varied considerably also by age, with the highest rates in the age group 20‐39 (Figure 2, Table S1). When comparing age‐specific risks of being diagnosed with influenza in primary care, this age group also had the highest risk ratio in most influenza seasons (Figure 3, Table S3).

### 3.2 Influenza burden in hospitals

**Figure 4:**
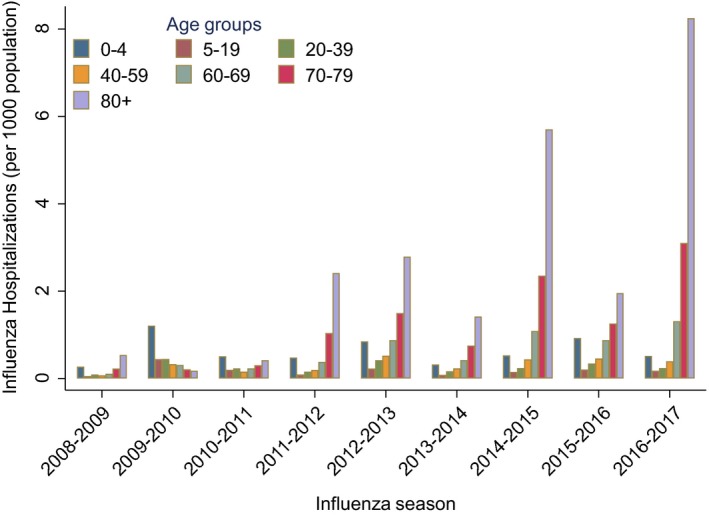
Seasonal age‐specific rates of hospitalized patients (per 1000 population) in Norway during 2008‐2017

In total, 23 415 patients were hospitalized with influenza during the nine studied influenza seasons. After excluding multiple hospital registrations in the same patients within a 120‐day interval, we included 22 228 patients for further analyses. Among the hospitalized patients, 52% were female. The average age was 56 years (median: 63). On average, 2470 patients were hospitalized per season (range: 579‐4973), and the average seasonal cumulative rate was 48 hospitalizations per 100 000 population (Table 1, Table S2). The highest rate of hospitalizations was seen in the 2016‐2017 season with 95 per 100 000 population. Over the period studied, there was a marked increase in the rate of hospitalizations among people 70 years and older (Figure 4).

**Figure 5:**
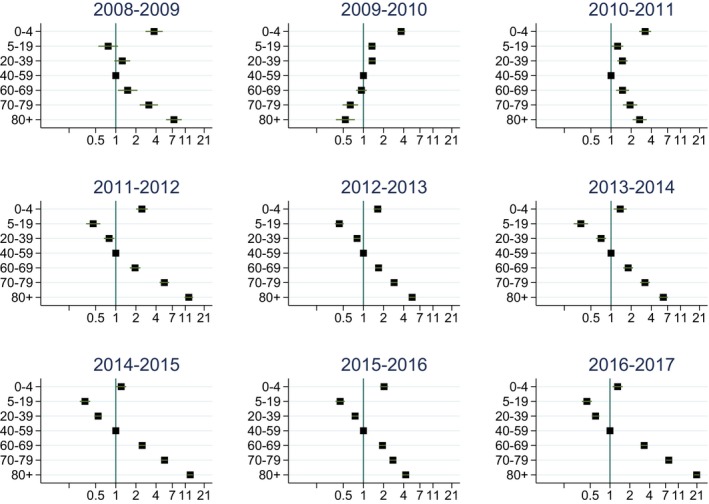
Risk ratios (with 95% confidence intervals) for being diagnosed with influenza in hospital care by age group in each influenza season. Logarithmic scale on *x*‐axis. Reference age group: 40‐59 years

In most seasons, influenza rates were highest for people above 70 years and above (Figure 4, Table S2). Children under 5 years of age had a higher risk of hospitalization compared to all other age groups up to 59 years (Figure 5, Table S3). The highest age‐specific cumulative incidence rate was seen in those above 80 years during the 2016‐17 season with 825 hospitalizations per 100 000 population (Table S2). In absolute numbers, however, the majority of hospitalized patients were in the age groups 5‐79 years, as this group accounts for larger part of the population.

Hospitalizations during the 2009‐2010 pandemic and the following season 2010‐2011 had a different age pattern than non‐pandemic outbreaks. During these two outbreaks, children below 5 years had the highest age‐specific hospitalization rates; the 2009‐2010 rates showed the highest rate in this age group during our entire study period with 121 hospitalizations per 100 000 population (Table S2).

From 2008 to 2017, patients with influenza in specialist care spent in total 130 855 days in hospital (Table 1), and 1318 patients had a hospital stay shorter than 24 hours. The mean number of days in hospital per patient was 5.4 (median 3 days). On average, influenza patients occupied 14 943 days of hospital beds each season.

### 3.3 Mortality

**Figure 6:**
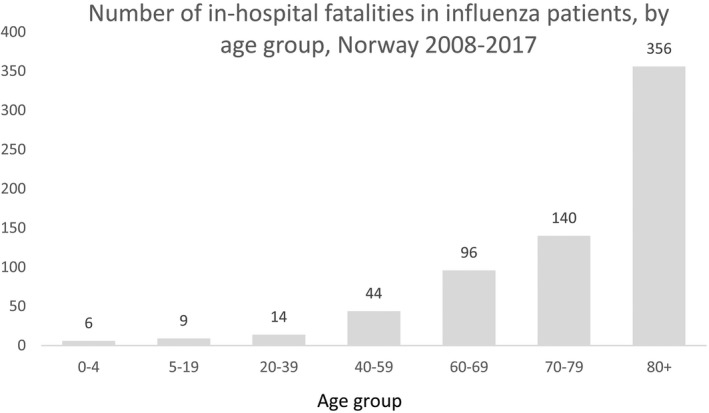
Fatal outcome in hospitalized patients diagnosed with influenza in different age groups in Norway, 2008‐2017

During the nine seasons in the study period, there were 665 fatalities registered among patients hospitalized with influenza. The average age at death in these patients was 76 years (median: 81). The number of fatalities varied from 8 in the 2008‐2009 season to 232 in the 2016‐2017 season (Table 1). When we excluded the 2009‐2010 pandemic outbreak, the average age of the fatal cases ranged from 49 years in the 2008‐2009 season to 82 years in the 2014‐2015 season. Most fatalities (91%) in non‐pandemic seasons were in patients from 60 years and older; however, there were fatal cases in children below 18 years of age every season, except in the 2011‐2012 season (Figure 6). During our study period, in total 18 influenza patients below 40 years of age died in hospital in regular seasonal outbreaks. During the 2009‐2010 pandemic outbreak, the average age of fatal cases was lower than in other seasons. Each season, between 1.4% and 4.7% of the hospitalized influenza patients died while in hospital, with a seasonal average of 3.0% (Table 1).

## 4 DISCUSSION

Our results show that influenza outbreaks cause a substantial disease burden in Norway. Each season, there are a substantial number of consultations in primary care, a considerable number of days in hospital care and numerous deaths. We observed an increasing trend in hospitalizations, both in terms of absolute numbers and rates. Hospitalizations and in‐hospital fatalities occurred in all age groups. The elderly and the very young had the highest hospitalization rates, except during the 2009‐2010 pandemic, in which the hospitalized and fatal cases were younger than in other influenza seasons.

On average, 1.7% of the population was diagnosed with influenza in primary care each influenza season, which is within the range of findings from other countries.^12^, ^25^, ^26^ In Norway, the public healthcare system offers primary and emergency outpatient care at low or no cost and hospital care is free for all citizens. This could result in a lower threshold for seeking medical help in Norway compared to other countries with higher personal costs for health care. The criteria for using the R80 influenza diagnosis include clinical symptoms or a combination of clinical symptoms, epidemiological data and laboratory confirmation.^22^ Even though laboratory confirmation is not required, the concurrent timing of hospital and primary care diagnoses supports that patients with a clinical picture coherent with influenza, and thus an influenza diagnosis, may have actual influenza infection. Still, some misclassification of influenza is expected as other respiratory agents may cause a similar clinical picture. In addition, patients with actual influenza infection may not have been diagnosed with influenza due to lack of testing, or they could be given another, more generic symptom‐based ICPC‐code like “cough” or “respiratory infection.”

We found on average 48 hospitalizations per 100 000 population during an influenza season, which is within the range found in other countries, but among the higher country‐specific rates.^4^, ^12^, ^27^, ^28^, ^29^ The increase in hospitalizations in the elderly could be a result of improved diagnostics in this group. From 2008 to 2017, the number of tests performed for influenza increased from approximately ten thousand a year to around 140 000.^18^ It is reasonable to believe that awareness of influenza increased after the 2009 pandemic. This may partly explain the increasing number of diagnosis over time. Another explanation could be that the threshold for hospitalizing the elderly has changed during the study period. The distribution of influenza viruses varies by season (Figure S1) and will most likely affect number and age distribution of patients.

Several influenza burden studies, like the one from Widgren and colleagues,^30^ include cases of pneumonia and other complications of influenza and find even higher incidence rates. Bacterial pneumonia is a well‐known complication of influenza infection, which contributes to the complete picture of the burden of severe influenza. We know from previous studies that chronic obstructive pulmonary disease, cardiovascular disorders and pregnancy are risk factors for complications due to an influenza infection and that influenza infection itself can cause cardiovascular and neurological disease.^31^, ^32^, ^33^, ^34^, ^35^ We did not include these consequences of influenza, and our estimation is a therefore lower than the total burden of influenza.

The hospitalization rate during the 2009‐2010 pandemic was lower than the overall average for our study period. This is similar to findings in other countries.^30^, ^36^ However, during the pandemic season, there was a marked difference in the age groups affected by severe influenza compared with regular influenza seasons. During the pandemic, the rate of hospitalization in the 0‐4 year age group was the highest recorded in the study period. The average age of the fatal cases in 2009‐2010 was 46 years which was much lower than the seasonal average of 80 years. These findings are in line with the age pattern previously seen in pandemic outbreaks.^15^

We found clear age differences among influenza patients in primary care and in hospitals. The relatively high rates of primary care consultations seen among adults in their working years may be explained by the need for doctor certificates for reimbursement of sick leave from work. Similarly, the increase in primary care patients in the age group 5‐19 in the 2016‐2017 season could be explained by stricter requirements implemented in 2016 for documentation of school absence for the 16‐ to 18‐year‐olds, when a doctor certificate requirement was introduced for the absence.^37^ Several studies 8, 12, 26, 38 have shown that children are more susceptible to influenza infection, but this is not reflected in our primary care consultation data. One explanation could be that most children have mild disease and not brought to the doctor. In addition, children might receive other diagnoses than influenza, since the clinical picture can resemble a range of other respiratory infections, which are frequent in younger age groups.

The low consultation rates in primary care and high hospitalization rates among the elderly could be explained by actual lower infection rate due to higher immunity, or that the elderly experience fewer symptoms and hence do not seek medical advice before the infection becomes severe or causes complications. The elderly might also have a higher threshold for seeking medical care and elderly who reside in nursing homes and are routinely cared for by other doctors than general practitioners and not captured in the national health registries.

### 4.1 Fatalities

As we included only deaths among hospitalized patients diagnosed with influenza, our study did not include all fatalities associated with influenza in Norway. The number of fatal cases found in our study is therefore lower than previous Norwegian estimates.^14^ Also, individual‐based methods are found to underestimate influenza mortality,^39^, ^40^ and models using all‐cause mortality in combination with influenza data are used in many European countries to make better estimations on total influenza‐related mortality.^41^ Patients dying from exacerbations of chronic disease or other complications after an influenza infection may not be registered as influenza‐related death. Patients might not be tested for influenza, or if they were tested, it was done too late in the course of disease to find a positive result. In addition, our study did not include data from nursing homes, where fatalities associated with influenza occur every season. A UK study estimated that only half of the fatal cases in those above 75 years happen in hospitals.^28^ When taking these factors into consideration, we assume that the actual mortality rates associated with influenza are considerable higher than numbers based on in‐hospital influenza diagnosis only.

### 4.2 Strengths and limitations

Our study includes complete follow‐up information on the entire Norwegian population for 10 years. We included data from all general practitioners and all hospitals in Norway from nine consecutive influenza seasons, thus potential bias from selection of participants and loss to follow‐up is unlikely. The independent collection in the different national registries reduces bias by potential dependency of diagnoses from the different sources.

However, the use of register data to study influenza does have limitations. The coding of influenza relies on the clinician's experience and practice of coding. Results from laboratory testing are not reported to the registries. However, in hospitalized patients, guidelines for most of the ICD‐10 codes require identification of influenza virus and tests for influenza are easily available with no restrictions on use. Studies indicate that the ICD‐codes tend to underestimate the number of influenza cases,^42^, ^43^, ^44^ however, the specificity of the ICD‐codes is shown to be high, indicating that the ICD‐codes of influenza are likely to reflect true influenza infection. In addition, many patients with influenza might not be tested for influenza or tested too late to confirm the diagnosis and may therefore be registered with other diagnoses. This could have contributed to a too low estimate of influenza in our study.

For general practitioners, laboratory confirmation is not required when using the ICPC diagnosis “R 80 Influenza.” This might lead to both under‐ and overestimation of numbers of the number of patients. In addition to medically attended influenza, there will be a large number of influenza patients who never seek medical care. A Belgian study found that around half of those experiencing influenza‐like illness seeks medical counselling.^45^ Simulation studies from the pandemic in Norway support this finding, as an estimated 30% of the population was infected with the pandemic influenza virus, while less than 5% of the population consulted a doctor.^46^ Considering this, the impact on society caused by influenza infections is higher than observed in our study.

## 5 CONCLUSION

Annual influenza outbreaks cause substantial morbidity and mortality and have a high impact on healthcare services. We found that the youngest and oldest are at highest risk of hospitalization during seasonal influenza outbreaks. Even though the elderly have the highest risk of dying, fatal outcome in young influenza patients is seen almost every season. The 2009‐2010 pandemic caused similar number of hospitalizations as seasonal outbreaks, but the hospitalized and fatal cases were younger compared to seasonal outbreaks.
