# Supplementary material for: Burden of medically attended influenza in Norway 2008‐2017
Source: Influenza Other Respir Viruses. 2019 Jan 13;13(3):240–7. doi: 10.1111/irv.12627 (PMC6468058; doi:10.1111/irv.12627)
Supplement: Supplementary file 1 [file IRV-13-240-s001.docx]

# Supplementary material

Table 1. Numbers and rates (per 100 000 population) of influenza diagnoses in primary care patients, by age groups and season in Norway, 2008-17

| Age group | 2008-2009 | 2009-2010 | 2010-2011 | 2011-2012 | 2012-2013 | 2013-2014 | 2014-2015 | 2015-2016 | 2016-2017 | Average rate |
| --- | --- | --- | --- | --- | --- | --- | --- | --- | --- | --- |
| **0-4** | 2506 (840) | 13367 (4398) | 3400 (1101) | 3423 (1101) | 4830 (1542) | 1700 (545) | 2828 (916) | 3307 (1083) | 2736 (902) | 1381 |
| **5-19** | 6826 (732) | 44531 (4758) | 14349 (1532) | 9621 (1026) | 14494 (1542) | 5058 (535) | 10533 (1108) | 10339 (1082) | 16415 (1711) | 1559 |
| **20-39** | 29060 (2272) | 72069 (5586) | 32761 (2503) | 28862 (2170) | 38598 (2857) | 19682 (1438) | 30689 (2215) | 29862 (2135) | 29616 (2099) | 2586 |
| **40-59** | 23011 (1775) | 47467 (3611) | 22119 (1661) | 25233 (1865) | 37803 (2756) | 17309 (1249) | 33891 (2422) | 27392 (1945) | 29527 (2091) | 2153 |
| **60-69** | 5052 (1040) | 8448 (1679) | 5195 (1001) | 6002 (1121) | 8876 (1618) | 4035 (721) | 9050 (1601) | 6851 (1201) | 8681 (1521) | 1278 |
| **70-79** | 1462 (509) | 2656 (914) | 1492 (507) | 1822 (613) | 2271 (744) | 1131 (356) | 2825 (840) | 2064 (580) | 3525 (927) | 666 |
| **80+** | 1002 (456) | 1337 (608) | 756 (342) | 1078 (486) | 1325 (598) | 595 (270) | 1561 (708) | 956 (434) | 1962 (888) | 532 |
| **Seasonal cumulative number (rate)** | 68919 (1436) | 189875 (3908) | 80011 (1627) | 76041 (1525) | 108197 (2142) | 49510 (969) | 91377 (1769) | 80771 (1549) | 92462 (1758) | 837224 (1696) |

Table 2. Numbers and rates (per 100 000 population) of influenza diagnoses in hospital care patients, by age groups and season in Norway, 2008-17

| **Age group** | **2008-2009** | **2009-2010** | **2010-2011** | **2011-2012** | **2012-2013** | **2013-2014** | **2014-2015** | **2015-2016** | **2016-2017** | **Average rate** |
| --- | --- | --- | --- | --- | --- | --- | --- | --- | --- | --- |
| **0-4** | 81 (27) | 368 (121) | 157 (51) | 149 (48) | 267 (85) | 100 (32) | 163 (53) | 283 (93) | 157 (52) | 62 |
| **5-19** | 52 (6) | 415 (44) | 184 (20) | 84 (9) | 213 (23) | 78 (8) | 144 (15) | 195 (20) | 169 (18) | 18 |
| **20-39** | 116 (9) | 576 (45) | 304 (23) | 206 (15) | 564 (42) | 227 (17) | 331 (24) | 478 (34) | 337 (24) | 26 |
| **40-59** | 94 (7) | 434 (33) | 209 (16) | 264 (20) | 714 (52) | 324 (23) | 614 (44) | 641 (46) | 559 (40) | 31 |
| **60-69** | 53 (11) | 155 (31) | 121 (23) | 203 (38) | 481 (88) | 236 (42) | 615 (109) | 500 (88) | 748 (131) | 62 |
| **70-79** | 66 (23) | 61 (21) | 89 (30) | 309 (104) | 457 (150) | 239 (75) | 791 (235) | 448 (126) | 1180 (310) | 119 |
| **80+** | 118 (54) | 39 (18) | 93 (42) | 535 (241) | 618 (279) | 313 (142) | 1257 (570) | 430 (195) | 1823 (825) | 241 |
| **Seasonal cumulative number (rate)** | 579 (12) | 2048 (42) | 1157 (24) | 1750 (35) | 3314 (66) | 1517 (30) | 3915 (76) | 2975 (57) | 4973 (95) | 2470 (48) |

Figure 1. Distribution of influenza viruses detected in the Norwegian sentinel surveillance for influenza, 2008-17.

Table 3. Risk ratios (RR) with confidence intervals (CI) for being diagnosed with influenza in primary and hospital care in each influenza season, by age group. Age group 40-59 is used as reference.

| **Primary care** | | | | | | | | | |
| --- | --- | --- | --- | --- | --- | --- | --- | --- | --- |
| **Season/**  **Age group** | **2008-09**  **RR (95% CI)** | **2009-10**  **RR (95% CI)** | **2010-11**  **RR (95% CI)** | **2011-12**  **RR (95% CI)** | **2012-13**  **RR (95% CI)** | **2013-14**  **RR (95% CI)** | **2014-15**  **RR (95% CI)** | **2015-16**  **RR (95% CI)** | **2016-17**  **RR (95% CI)** |
| **0-4** | 0.47 (0.45-0.49) | 1.22 (1.20-1.24) | 0.66 (0.64-0.69) | 0.59 (0.57-0.61) | 0.56 (0.54-0.58) | 0.44 (0.42-0.46) | 0.38 (0.36-0.39) | 0.56 (0.54-0.58) | 0.43 (0.42-0.45) |
| **5-19** | 0.41 (0.40-0.42) | 1.32 (1.30-1.34) | 0.92 (0.90-0.94) | 0.55 (0.54-0.56) | 0.56 (0.55-0.57) | 0.43 (0.42-0.44) | 0.46 (0.45-0.47) | 0.56 (0.54-0.57) | 0.82 (0.80-0.83) |
| **20-39** | 1.28 (1.26-1.30) | 1.55 (1.53-1.57) | 1.51 (1.48-1.53) | 1.16 (1.14-1.18) | 1.04 (1.02-1.05) | 1.15 (1.13-1.17) | 0.91 (0.90-0.93) | 1.10 (1.08-1.12) | 1.00 (0.99-1.02) |
| **40-59** | Reference group | Reference group | Reference group | Reference group | Reference group | Reference group | Reference group | Reference group | Reference group |
| **60-69** | 0.59 (0.57-0.60) | 0.47 (0.45-0.48) | 0.60 (0.58-0.62) | 0.60 (0.59-0.62) | 0.59 (0.57-0.60) | 0.58 (0.56-0.60) | 0.66 (0.65-0.68) | 0.62 (0.60-0.63) | 0.73 (0.71-0.75) |
| **70-79** | 0.29 (0.27-0.30) | 0.25 (0.24-0.26) | 0.31 (0.29-0.32) | 0.33 (0.31-0.35) | 0.27 (0.26-0.28) | 0.29 (0.27-0.30) | 0.35 (0.33-0.36) | 0.30 (0.29-0.31) | 0.44 (0.43-0.46) |
| **80+** | 0.26 (0.24-0.27) | 0.17 (0.16-0.18) | 0.21 (0.19-0.22) | 0.26 (0.25-0.28) | 0.22 (0.21-0.23) | 0.22 (0.20-0.23) | 0.29 (0.28-0.31) | 0.22 (0.21-0.24) | 0.43 (0.41-0.45) |
| **Hospital care** | | | | | | | | | |
| **0-4** | 3.74 (2.78-5.04) | 3.67 (3.19-4.21) | 3.24 (2.63-3.98) | 2.46 (2.01-3.00) | 1.64 (1.42-1.89) | 1.37 (1.10-1.72) | 1.20 (1.01-1.43) | 2.04 (1.77-2.34) | 1.31 (1.10-1.56) |
| **5-19** | 0.77 (0.55-1.08) | 1.34 (1.17-1.54) | 1.25 (1.03-1.53) | 0.46 (0.36-0.59) | 0.44 (0.37-0.51) | 0.35 (0.28-0.45) | 0.35 (0.29-0.41) | 0.45 (0.38-.0.53) | 0.45 (0.38-0.53) |
| **20-39** | 1.25 (0.95-1.64) | 1.35 (1.19-1.53) | 1.48 (1.24-1.77) | 0.79 (0.66-0.95) | 0.80 (0.72-0.90) | 0.71 (0.60-0.84) | 0.54 (0.48-0.62) | 0.75 (0.67-0.85) | 0.60 (0.53-0.69) |
| **40-59** | Reference group | Reference group | Reference group | Reference group | Reference group | Reference group | Reference group | Reference group | Reference group |
| **60-69** | 1.51 (1.08-2.11) | 0.93 (0.78-1.21) | 1.49 (1.19-1.86) | 1.94 (1.62-2.33) | 1.68 (1.50-1.89) | 1.80 (1.53-2.13) | 2.48 (2.22-2.77) | 1.93 (1.71-2.17) | 3.31 (2.97-3.70) |
| **70-79** | 3.12 (2.28-4.28) | 0.64 (0.49-0.83) | 1.93 (1.50-2.47) | 5.33 (4.52-6.28) | 2.88 (2.56-3.24) | 3.22 (2.72-3.80) | 5.36 (4.83-5.96) | 2.77 (2.45-3.12) | 7.84 (7.09-8.67) |
| **80+** | 7.41 (5.65-9.72) | 0.54 (0.39-0.75) | 2.68 (2.10-3.42) | 12.38 (10.68-14.34) | 5.36 (4.81-5.97) | 6.06 (5.19-7.08) | 13.00 (11.80-14.31) | 4.29 (3.80-4.85) | 20.84 (18.96-22.91) |
